# Electrolyte-Gated Organic Field-Effect Transistors for Quantitative Monitoring of the Molecular Dynamics of Crystallization at the Solid–Liquid Interface

**DOI:** 10.1021/acs.nanolett.1c04424

**Published:** 2022-03-24

**Authors:** Jincheng Tong, Amadou Doumbia, Raja U. Khan, Aiman Rahmanudin, Michael L. Turner, Cinzia Casiraghi

**Affiliations:** Department of Chemistry, University of Manchester, Manchester M13 9PL, United Kingdom

**Keywords:** Electrolyte-gated organic field-effect transistor, crystallization, electrical double layer, molecular dynamics, solid−liquid interface

## Abstract

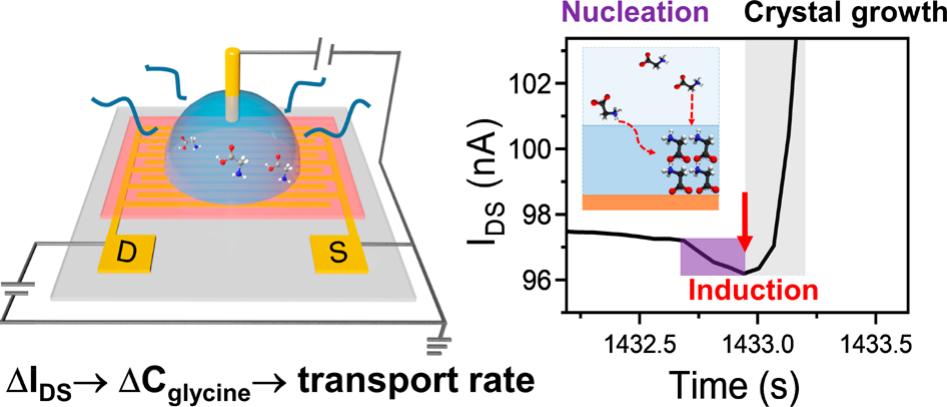

Quantitative measurements of molecular dynamics at the solid–liquid interface are of crucial importance in a wide range of fields, such as heterogeneous catalysis, energy storage, nanofluidics, biosensing, and crystallization. In particular, the molecular dynamics associated with nucleation and crystal growth is very challenging to study because of the poor sensitivity or limited spatial/temporal resolution of the most widely used analytical techniques. We demonstrate that electrolyte-gated organic field-effect transistors (EGOFETs) are able to monitor in real-time the crystallization process in an evaporating droplet. The high sensitivity of these devices at the solid–liquid interface, through the electrical double layer and signal amplification, enables the quantification of changes in solute concentration over time and the transport rate of molecules at the solid–liquid interface during crystallization. Our results show that EGOFETs offer a highly sensitive and powerful, yet simple approach to investigate the molecular dynamics of compounds crystallizing from water.

Crystallization from solution is a fundamental process observed in nature as well as a critical component of many industrial processes.^1−5^ The rearrangement of the molecules from solution into a solid phase can be achieved through evaporation or cooling.^6^ The former is the most popular method of crystallization, as it relies only on solvent evaporation at constant temperature.^6^ In particular, evaporating droplets are often used to study crystallization^7−10^ as they offer a simple and well-defined system. Furthermore, evaporation of small droplets is crucial in many processes, ranging from inkjet printing^11,12^ and spray coating^13^ to pharmaceuticals^14^ and environmental science.^15^

A detailed understanding of the nucleation and crystal growth processes is still lacking, leading to challenges in crystal engineering and polymorphism control.^16−19^ Getting insights into the dynamics of the crystallization process is very challenging because this typically takes place across a broad range of length and time scales. Most of the common characterization techniques do not have either a high enough sensitivity or the required spatial/temporal resolution to quantify the changes in the molecular assembly over the time of crystallization.^5,16,20,21^ Often, these techniques provide information only on the phenomena happening in the bulk, while in many cases, like in an evaporating droplet on a surface, nucleation is usually heterogeneous, so techniques that are highly sensitive to changes at the solid–liquid interface need to be employed. Importantly, the techniques used to study crystallization need to be non-invasive, as additional surfaces in contact with the molecules can influence nucleation and crystal growth.^18,22−24^

In this framework, technologies used for sensing are extremely attractive because these devices are typically designed to achieve high sensitivity and fast response time.^25−29^ We recently demonstrated the use of an interdigitated electrode array to study the crystallization dynamics of small organic molecules from evaporative droplets, achieving a temporal resolution of 15 ms.^30^ However, in these devices the recorded current was very low (max 300 pA) and comparable to the noise level, leading to only qualitative information on the droplet crystallization process dynamics.

A simple way to achieve signal amplification is to move from a simple electrode array approach to a transistor, where any capacitance modification is transduced into an output current variation that is several orders of magnitude higher than that typically associated with the electrode array method.^31,32^ Because crystallization happens in solution, electrolyte-gated organic field-effect transistors (EGOFETs) offer an attractive solution to achieve real-time monitoring of the crystallization process and deliver inherent signal amplification in the device to enable quantitative information to be obtained on the nanoscale processes happening at the solid–liquid interface during crystallization. In an EGOFET, upon the application of a gate voltage, electrical double layers are formed at the gate electrode/electrolyte and semiconductor/electrolyte interfaces. These electrical double layers have thickness in the range of few nanometers and can reach a very high capacitance (in the range of a few μF), making the electrical response of the device very sensitive to phenomena happening at the solid–liquid interface.^31,32^

In this work, we exploit EGOFETs for real-time monitoring of nucleation and crystal growth from an evaporative droplet. Traditionally, EGOFETs are used in static conditions; that is, the analyte concentration is measured at a fixed point in time, typically after the device reaches equilibrium, and there are no molecular changes taking place in solution.^26,28,29^ In our approach, we measure the change in current over time while an aqueous droplet of glycine solution is evaporating. We demonstrate that changes in the recorded current can be assigned to changes in the concentration at the electrical double layer, as a result of the heterogeneous crystallization process, hence providing values of the molecular transport rate at different stages of crystallization, which has not been reported to date. Our results are of fundamental importance in the study of crystallization as they provide quantitative insights into the crystallization dynamics during droplet evaporation and demonstrate the major potential of EGOFETs in crystallization studies, which is well beyond their traditional use in biosensing.

## Results and Discussion

We use glycine as the reference molecule as its crystallization from evaporative droplets has been widely studied.^23,24,33^ In contrast to traditional sensing by EGOFET, in our case the droplet is evaporating, so one has to take into account that several processes, i.e., crystal surface coverage, water evaporation, and droplet shrinking, are all happening simultaneously, while glycine molecules will concentrate at the solid–liquid interface and will start crystallizing. Because of this, experiments from evaporative droplets containing water only have been performed and used as a control sample. The crystallization process takes 20–30 min under our experimental conditions, so one has also to make sure that the semiconducting channel is not affected by the presence of the glycine molecules over this relatively long time.

### Control Experiments

**Figure 1:**
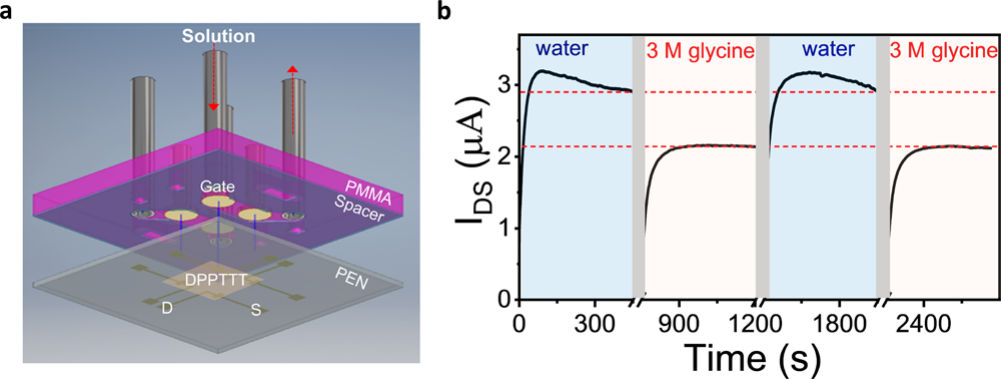
EGOFET integrated with a microfluidic system and stability test. (a) Schematic of the EGOFET integrated with the microfluidic system to test the stability of the device. The interdigitated gold drain–source electrodes are patterned on the flexible PEN substrate; the organic semiconductor of DPPTTT is spin-coated on top of the electrodes; PMMA is used to integrate the gold wire and microfluidic tubes; an adhesive spacer is used to connect and confine the cell of the device; solution can be injected by syringe pump into the cell to complete the EGOFET. Holes in the PMMA and spacer align well with the pads of the drain and source electrodes to allow them to connect with probe station for characterization. (b) The *I*_DS_ curves measured by sequentially pumping into the microfluidic system water, 3 M glycine solution, water and 3 M glycine solution under a constant *V*_G_ of −0.8 V and *V*_DS_ of −0.7 V. The gray parts between water and 3 M glycine solution represent the periods where the new solution was pumped into the cell for 5 min to wash out and replace the previous solution.

To evaluate the stability of the EGOFET device in the experimental time frame, we have connected the device to a microfluidic system (Figure 1a; more details in section S1) because it does not allow evaporation of water, enabling the study of the characteristics of the device under extended time scales.

Water and 1 M glycine solution were sequentially injected into the microfluidic channel by using a syringe pump with a flow rate of 100 μL/min. The electrical characteristics show little change for up to 30 consecutive cycles of measurement, lasting over 4260 s (Figure S1a,b). Devices were stored for 12 h in 1 M glycine solution and then characterized for another 20 consecutive cycles (1540 s in total), giving essentially identical transfer curves (Figure S1c). The stability of the fabricated EGOFET was further confirmed by continuous measurements (Figure S1d).

To exclude any diffusion of glycine into the semiconductor, water and 3 M glycine were sequentially injected twice into the microfluidic channel. Between each measurement, the microfluidic channel was washed for 5 min to remove the previous solution from the device. As shown in Figure 1b, the drain–source current (*I*_DS_) recovers to its original level once water or 3 M glycine is injected into the microfluidic system. The decrease of the current obtained using 3 M glycine as the electrolyte can be ascribed to the change in capacitance and the concomitant shift of the threshold voltage (Figure S2), showing that the device is sensitive to the presence of glycine. It is also noteworthy that the output current of these devices is a few microamperes, several orders of magnitude higher than the one observed with microelectrode arrays,^30^ confirming the inherent EGOFET signal amplification.

### Calibration Curve

**Figure 2:**
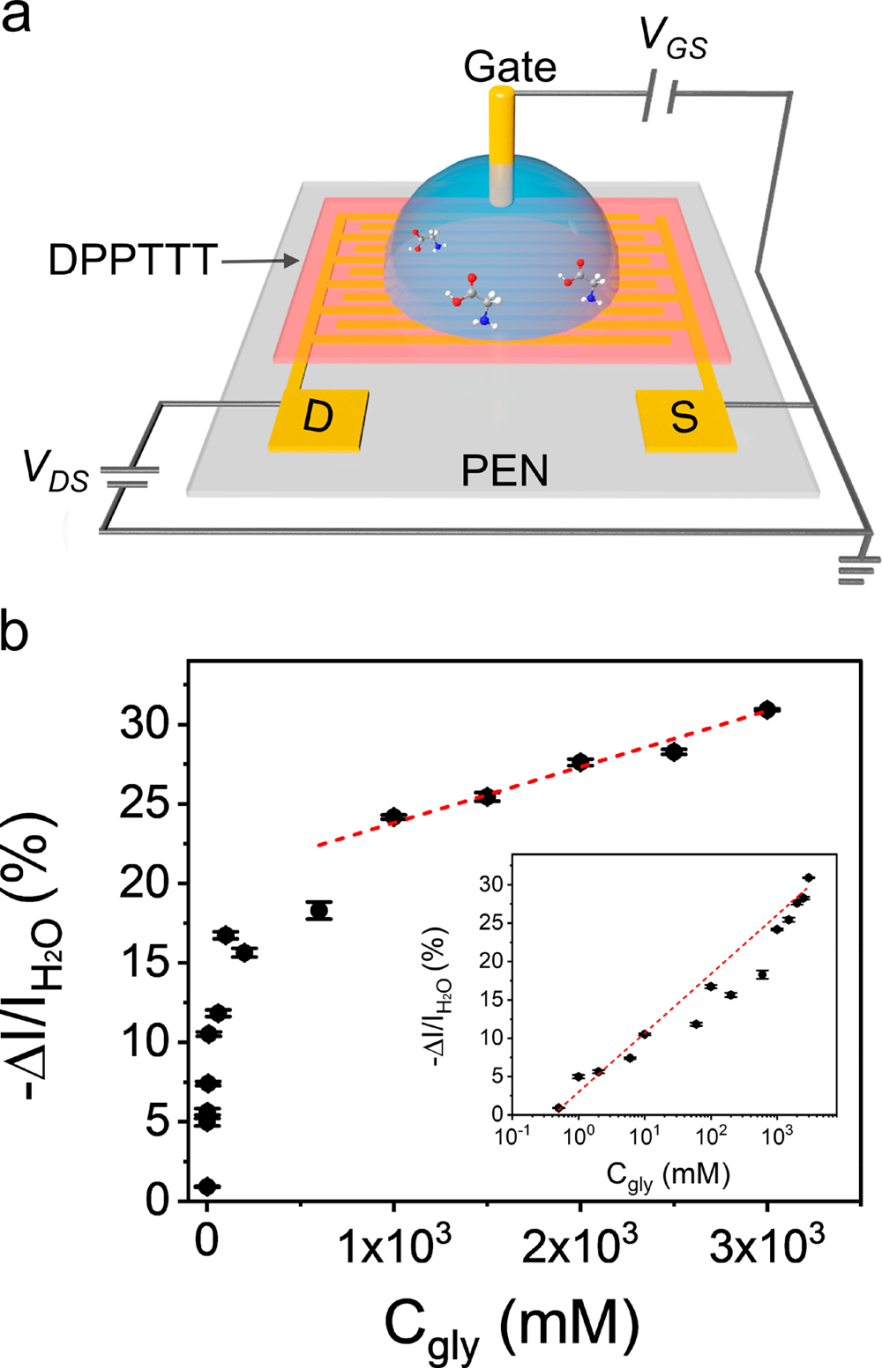
EGOFET setup applied to an evaporative droplet. (a) Schematic of the device and experimental setup. Glycine aqueous solution is drop-casted on top of the organic semiconductor layer (DPPTTT), and the gate of gold wire is immersed into the droplet to complete the device. The open system allows the evaporation of the droplet to induce crystallization. (b) Calibration curve of the EGOFET response (−Δ*I*/*I*_H_2_O_) as a function of *C*_gly_ obtained at *V*_G_ of −0.8 V and *V*_DS_ of −0.7 V. The red dashed line is the linear fitting of the data above 1 M concentration and for the entire concentration range in the semilogarithmic format (inset).

An open system, making use of an evaporative droplet (Figure 2a), was employed to enable nucleation to always start at the contact region of the droplet (Figure S4 and Movie S1) and to measure the device sensitivity. The molecular transport and rearrangement caused by nucleation will cause changes in the electrical double layer, hence causing a change in the current, from which one can extract the molecule concentration at the electrical double layers (the EGOFET functioning principles are given in section S2). The microfluidic setup is not suitable for such measurements because crystallization would happen in random locations and times, while the device is sensitive only at the liquid–solid interface, hence making it impossible to establish any correlation between concentration and changes in the observed current. Complete details on the setup are given in section S1.

The measurements were performed by placing a droplet of solution containing glycine at different concentrations (up to 3 M) over the active channel under ambient conditions. A Faraday cage was used to minimize electrical noise.^34^ Representative transfer and output curves obtained using water and glycine solution are shown in Figure S5. For the calibration, the current was measured for different glycine concentrations after the device operation was stabilized, i.e., at around 70 s after positioning of the droplet (Figure S6). Before each glycine concentration measurement, water was used to rinse the device and the signal from pure water was measured. The relative change in the current measured at a given glycine concentration with respect to that measured for water only (−Δ*I*/*I*_H_2_O_) was then calculated and plotted as a function of the glycine concentration. Figure 2b shows a decrease in current upon increasing of the glycine concentration. In particular, we note that at low concentrations (<1 M) the change in current scales linearly with the logarithm of the concentration. This behavior can be ascribed to the screening effects of glycine ions on the electrical double layer due to their large dipole moments and electrostatical interaction with water, as observed for other ions.^35,36^ A linear fitting of the data (red dashed line in inset Figure 2b) shows that a change in the current of 8% corresponds to 1 order of magnitude change in concentration over the range investigated. At concentrations above 1 M, which are of interest for our crystallization experiments, the relative change in current scales linearly with the glycine concentration (equations provided in sections S4.2 and S5.2); hence, a decrease in current is directly proportional to an increase in the concentration of molecules at the solid–liquid interface and vice versa, and this change can be quantified by using the equations in section S5.2.

### Real-Time Monitoring of Crystallization Using an EGOFET Device

The measurements were carried out by recording drain–source current and gate current at *V*_G_ of −0.8 V and *V*_DS_ of −0.7 V during the evaporation of the droplet. The measurement time started from the deposition of the droplet on the channel until complete water evaporation. The time interval between measurements was set at 50 ms, the smallest value that can be used in the experimental setup.

**Figure 3:**
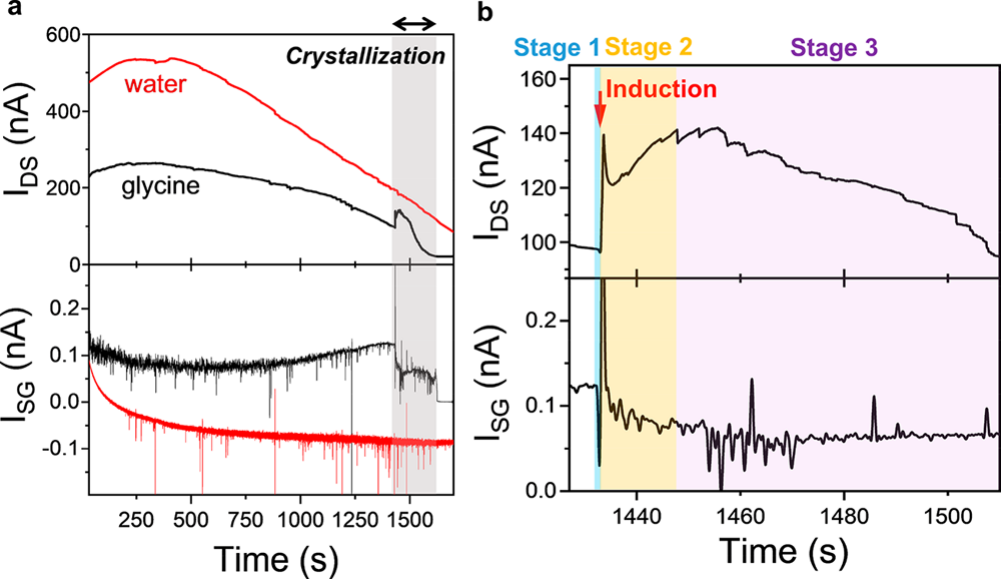
Real-time monitoring of the crystallization of glycine in water. (a) Changes in *I*_DS_ and *I*_SG_ over time after drop casting of water or 1 M glycine solution at a fixed *V*_G_ (−0.8 V) and *V*_DS_ (−0.7 V). A clear discontinuity in *I*_DS_ is observed in the case of glycine, upon crystallization. Note that I_SG_ of water has been scaled down of 0.105 nA to enable better comparison with I_SG_ from the glycine solution. (b) Enlarged view related to the changes associated with crystallization. The numbers 1, 2, and 3 identify the different crystallization stages.

**Figure 4:**
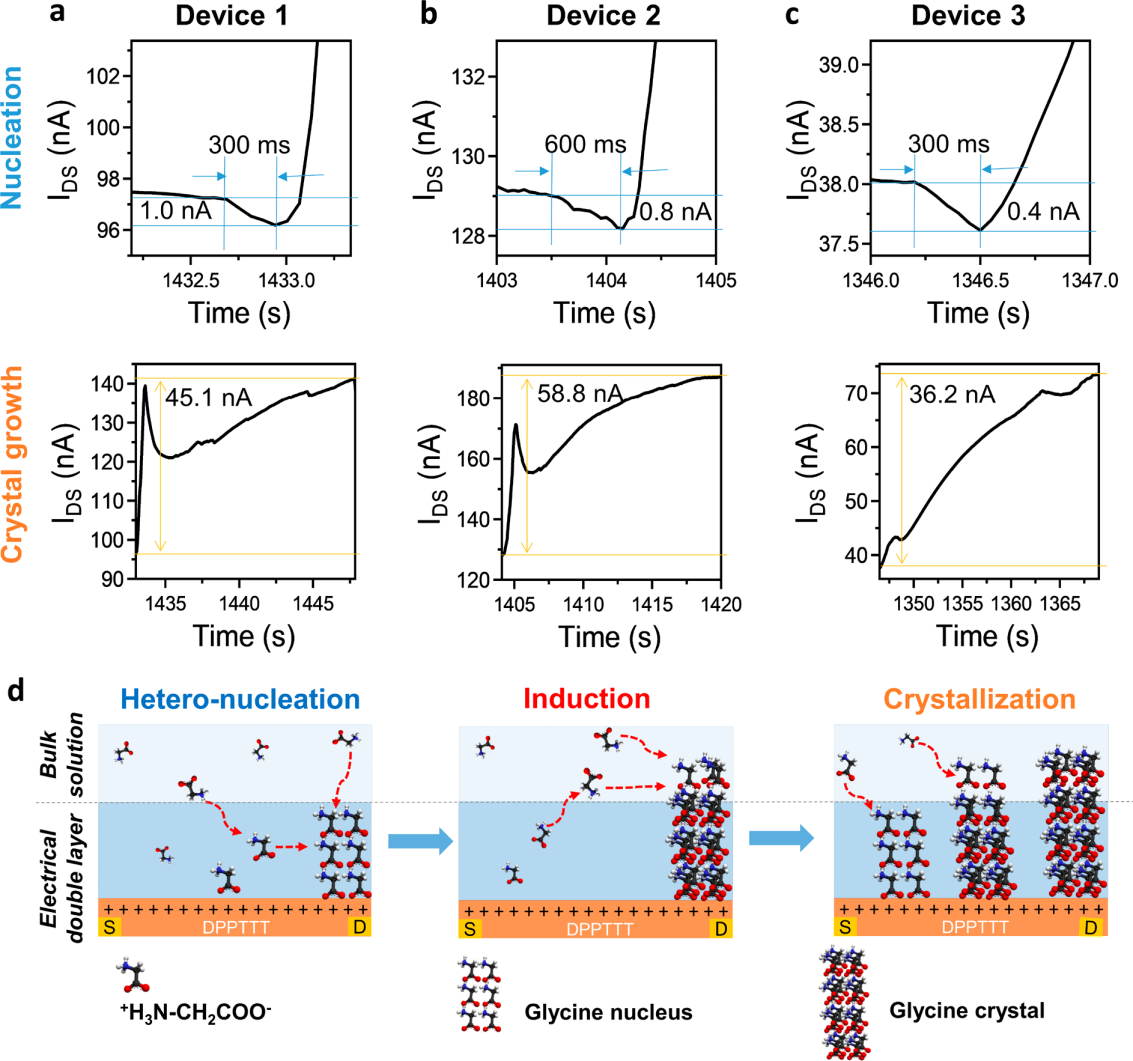
Nucleation and crystal growth stages revealed by EGOFET measurement. (a–c) Enlarged view of the observed stage 1 (nucleation) and stage 2 (crystal growth) based on the real-time monitoring of glycine crystallization by EGOFET over three devices. The values inserted into the figures show the current change over the period for nucleation and crystal growth. (d) Schematic illustration of the mass transport of the glycine molecules close to the solid–liquid interface during heterogeneous nucleation, induction, and crystal growth. The red dashed arrows show the molecular transport direction in each stage.

Control experiments were conducted with pure water (Figure 3a): the measured drain–source current (I_DS_) reaches a steady state at around 500 s and then decreases slowly, almost linearly, reaching the off state after 1821 s. The reduction of the measured current is due to the decrease in contact area, caused by the droplet shrinking, in agreement with previous experiments using interdigitated electrodes.^30^ A decrease in current due to solvent evaporation is also observed upon evaporation of a droplet containing 1 M glycine (Figure 3a). However, in this experiment a sharp increase in current is observed after 1433 s. As formation of crystals become visible at this time, this sharp increase in the current is assigned to crystal induction, Figure 3b, in agreement with our previous work.^30^ The relative increase of the current is 46.9% (Figure 4a and Table S3). By using the calibration curve in Figure 2b, this change is equivalent with a decrease of approximately 6 orders of magnitude in the glycine concentration in the electrical double layer. A discontinuity in gate current is also observed (see red arrow in Figure 3b) at the induction time; however, the magnitude of this change is impossible to quantify due to the large noise in the gate current.

To note that crystals produced during the crystallization may cover some part of the semiconductor layer, resulting in a decrease of the active area; hence, this effect may contribute to a decrease in the current. However, at the induction time, we observed a sharp and strong increase of the current, while a decrease in the active area should cause the current to decrease. Furthermore, due to the low evaporation rate and the fast crystallization dynamics, no detectable change in the area of the droplet was observed at the crystal’s induction time (see Figure S4 and Movie S1). Therefore, the sharp increase in the current is largely attributed to the crystallization process.

After the sudden increase, the current slowly decreases until reaching the off state (Figure 3a). This is due to the reduction in droplet size during evaporation and the growth of new crystals on the semiconductor. Remarkably, the current remains of the order of tens to hundreds of nanoamperes over the whole process, Figure 3b, indicating that the device operates effectively as an EGOFET before, during, and after crystal induction. Indeed, the EGOFET device performance can recover back to the original level after washing of the crystals from the channel, confirming that the presence of the crystals on the semiconductor does not affect the device readout (see section S4.4).

Overall, our results confirm that the electrical readout of an EGOFET can be used to precisely identify the induction time, which in addition to the measured evaporation rate of water (see section S5 and Tables S1 and S2 for details) allows us to get a supersaturation ratio of 1.3 ± 0.2 for 1 M glycine solution. This value is in good agreement with the one obtained using an electrode array (1.24 ± 0.12).^30^

Our results indicate that the changes in the current upon solvent evaporation of a droplet of glycine solution can be ascribed to three stages: a small and sharp dip in the current over 300–600 ms before induction (stage 1); a peak in the current associated with the induction time, followed by a slow increase in current over tens of seconds (stage 2); and a slow decrease in the current, characterized by small fluctuations until complete crystallization (stage 3). In contrast to the microarray approach, the enhanced sensitivity of the EGOFET allows us to quantify the changes in molecule concentration at the liquid–solid interface happening during crystallization from the changes in current by using the calibration curve (Figure 2b), hence providing quantitative information on the transport rates associated with nucleation and crystal growth, which has not been reported to date. In the case of stage 1, the average decrease in current measured on three devices is ∼0.9%, corresponding to an average increase of concentration in the electrical double layer of ∼25.5% and an average transportation rate for stage 1 (*R*_1_) of ∼3.2 M s^–1^ (Table S3). For stage 2, an average increase in current of ∼63.0% was observed. This value corresponds to loss of almost all of the glycine molecules in the electrical double layer during stage 2 as most of the molecules are used to grow the crystals. Note that the calibration curve was measured only up to a minimum concentration of 0.5 mM glycine (Figure 2b), so exact quantification of glycine concentrations below this value is not possible. However, it is clear that the remaining local concentration of molecules at the end of stage 2 is very low and can be approximated to zero to calculate the transport rate at stage 2 (*R*_2_), which is found to be ∼0.3 M s^–1^ (Table S3). This is about 1 order of magnitude lower than *R*_1_, implying that crystal growth leads to slower changes in the electrical double layer when compared to nucleation, even though a larger number of molecules in the whole solution are used for the crystal growth. In other words, at the end of stage 2, the concentration in the electrical double layer is below the saturation concentration, meaning that most of the molecules in the electrical double layer are used to grow the crystals rather than forming new nuclei. Note that the device is still working as an EGOFET during and after stage 2, even if there are few glycine molecules left in the electrical double layer, because there is still water at the interface, as full evaporation is not reached yet. In stage 3, a slow decrease of the current is observed. This may be ascribed to the fact that the entire surface of the droplet has been mostly covered by the crystals at the end of stage 2 (Figure S4 and Movie S1); hence, the active area of the device is reduced. Additionally, the glycine concentration may increase again with decreasing of the active area, reaching supersaturation, which will also contribute to decreasing the current. Figure 4d shows a schematic of the processes happening during the experiment: initially, the molecules are uniformly distributed in the droplet; after some time, a local increase of molecules is detected by the EGOFET as a decrease in the measured current; that is, in stage 1 additional molecules are transported from the bulk into the electrical double layer (indicated by the red dashed arrows), leading to heterogeneous nucleation (stage 1). At stage 2 the current increase is correlated with a decrease in the number of molecules in the electrical double layer, as the nuclei formed in stage 1 grow by taking molecules from both the bulk solution and the electrical double layer. Stage 3 corresponds to crystal growth and possible further nucleation.

## Conclusions

In summary, we demonstrate that EGOFETs are powerful devices for the study of crystallization of water-based solutions, with stable operation over several hours of measurement. Through monitoring changes in the drain–source current, it is possible to quantify changes in the molecule concentration and the transport rates associated with nucleation and crystal growth of glycine at the solid–liquid interface. In principle, any solution that can work as an electrolyte and at the same time allows crystallization to happen under ambient conditions and in a relatively short time can be analyzed by using an EGOFET. Therefore, other crystalline compounds (e.g., small molecules, salts, and macromolecules) that are soluble in water can be studied with this approach, although the experimental conditions will need to be optimized for each case.

## Supporting Information Available

Methods and materials; fundamentals of the EGOFET; EGOFET coupled with the microfluidic device; electrical characterization of the EGOFET, calibration measurements, real time measurements, and stability of the EGOFET device over the crystallization process; calculation of the critical concentration and supersaturation ratio of glycine; calculation of molecule concentration and transfer rates during crystallization (PDF)
Movie of the real-time crystallization process (Movie S1) (MP4)
