# Supplementary material for: Electrolyte-Gated Organic Field-Effect Transistors for Quantitative Monitoring of the Molecular Dynamics of Crystallization at the Solid–Liquid Interface
Source: Nano Lett. 2022 Mar 24;22(7):2643–9. doi: 10.1021/acs.nanolett.1c04424 (PMC9098175; doi:10.1021/acs.nanolett.1c04424)
Supplement: Supplementary file 1 — nl1c04424_si_002.pdf [file nl1c04424_si_002.pdf]

Supporting Information:

EGOFETs for quantitative monitoring of the molecular dynamics of crystallization at the solid-liquid interface

Jincheng Tong[1],* Amadou Doumbia[1], Raja U. Khan[1], Aiman Rahmanudin[1], Michael L. Turner[1],* and Cinzia Casiraghi[1]*

[1]Department of Chemistry, University of Manchester, Manchester, M13 9PL, United Kingdom

* Email: tongjincheng@outlook.com (J.T.); michael.turner@manchester.ac.uk (M.L.T.); cinzia.casiraghi@manchester.ac.uk (C.C.)

Section S1. Methods and materials

Section S2. Fundamentals of the EGOFET

Section S3. EGOFET coupled with the microfluidic device

Section S4. Real-time crystallization study from evaporative droplets

Section S4.1. Electrical characterization of the EGOFET

Section S4.2. Calibration measurements

Section S4.3. Real-time measurements

Section S4.4. Stability of the EGOFET device over the crystallization process

Section S5. Calculations of the droplet crystallization dynamics

Section 5.1. Critical concentration and supersaturation ratio

Section 5.2. Molecule concentrations and transfer rates

Section S6. Movie S1

Section S1. Methods and materials

Materials

Poly[2,5-(2-octyldodecyl)-3,6-diketopyrrolopyrrole-alt-5,5-(2,5-di(thien-2-yl)thieno[3,2-b]thiophene)] (DPPTTT) with a molecular weight (M_n) around 30 kDa was purchased from Ossila. Pentafluorobenzenethiol (PFBT) and 1H,1H,2H,2H-perfluorodecanethiol (PFDT) were purchased from Sigma-Aldrich. Glycine was also purchased from Sigma-Aldrich and solutions were prepared by dissolving in pure water purchased from Fisher Scientific. Dichlorobenzene was used to dissolve DPPTTT, acetone and isopropanol (IPA) were used to wash the substrates.

Fabrication of the interdigitated source-drain electrodes

Interdigitated gold electrodes with a channel width of 1600 μm and a channel length of 60 μm were patterned on a flexible substrate by a combination of direct laser photoresist (S1805) lithography, metal evaporation and lift-off techniques. The substrate was a polyethylenenaphthalate (PEN) sheet laminated on glass. This was sonicated in IPA for 5 minutes, blown dry with a stream of nitrogen and coated with S1805 (Speed: 7000 rpm, 1000 rpm·s^{-1}, 60 s). The substrate was heated at 110 °C for 5 minutes to remove residual solvents and a pattern written by a 405 nm laser beam using a direct laser writer (mr-DWL, micro resist technology GmbH, Germany). The exposed substrate was than immersed in MF319 solution and then in water for 60 s each. This process removed the exposed photoresist and the water quenched the reaction of MF319 with S1805. The process was followed by coating the substrate with 5 nm of chromium and 40 nm of gold. Chromium was used to promote the adhesion of gold on the PEN substrate. Finally, the substrate was sonicated in acetone for a few minutes to lift-off the gold and produce the desired interdigitated electrode pattern.

Fabrication of the EGOFETs for *in-situ* measurements

The interdigitated electrodes were cleaned with UV/Ozone treatment for 15 min and then treated with PFBT (1 h incubation in 10 mM PFBT solution diluted in IPA) to decrease the injection barrier that exists between gold and DPPTTT[1]. After these treatments, a solution (8 mg/mL) of DPPTTT in DCB was spin coated onto the patterned source-drain electrodes. The obtained film was annealed at 140 °C for 40 min to fully remove the solvent. The gold wire with a diameter of 0.5 mm, used as top gate, was treated with PFDT (1 h incubation in 10 mM PFDT solution diluted in IPA). A droplet (2 µL) of water or different concentration of glycine solution was placed on the substrate and the PFDT treated gold wire was immersed into it for the measurements.

Integration with the microfluidic setup

A double-sided tape with microfluidic channels laser written into it was stuck on top of the source-drain substrate coated with DPPTTT as described above. An array of 1 mm^2 gold electrodes was patterned onto a second PEN substrate and this was attached to a poly(methyl methacrylate) carrier. This assembly was then stuck to the second surface of the double sided tape to close the device. Holes in the substrate of the gate electrode substrate allowed water or glycine solution to enter the microfluidic channels. A syringe pump was used to control the flow rate of the solution.

Electrical measurements

These were conducted by using an Agilent B1500A semiconductor parameter analyzer in conjunction with a custom made probe station. All measurements were performed in an ambient environment at room temperature. For the EGOFETs with microfluidic device, water or glycine solution was first pumped into the microfluidic channel with a flow rate of 100 µL min^{-1} for 5 minutes to ensure it fully covered the channel and to remove any contaminants in the system before starting the measurement.

Section S2. Fundamentals of the EGOFET

By applying a voltage between the source and drain terminates, the charges accumulated in the DPPTTT starts flowing, by generating I_{DS} as following:[2]

$$I_{\mathrm{DS}} = C_{\mathrm{i}}\mu(V_{\mathrm{G}} - V_{\mathrm{Th}})^2(W/2L) \quad (1)$$

where W is the channel width, L is the channel length, μ is the carrier mobility of the DPPTTT layer, C_i is the total capacitance of the EDLs, V_G is the gate voltage, V_{Th} is the threshold voltage. To extract the values of μC_i, and V_{Th}, the square root of the I_{DS} is plotted against V_G as:

$$\sqrt{I_{\mathrm{DS}}} = \sqrt{\frac{WC_{\mathrm{i}}\mu}{2L}}(V_{\mathrm{G}} - V_{\mathrm{Th}}) \quad (2)$$

μC_i can therefore be extracted by the slope of $(I_{DS})^{0.5}$ *vs* V_G by linear fitting of the data. The extrapolated intercept of this straight line with the V_G axis is taken as V_{Th}.

Section S3. EGOFET coupled with the microfluidic device

The transfer characteristics of the EGOFETs for both water (**Figure S1a**) and glycine solution (**Figure S1b**) display typical *p*-channel transistor responses, with a small current hysteresis and a high on/off ratio (10^3 - 10^4).

Figure S2 shows that the slope of the line obtained when plotting $(I_{DS})^{0.5}$ vs V_G (eq. 2) in the case of 3 M glycine decreases by 13.8% compared to water solution, while the intercept, which gives the threshold voltage (V_{Th}), is shifted to positive voltage of around 70 mV.

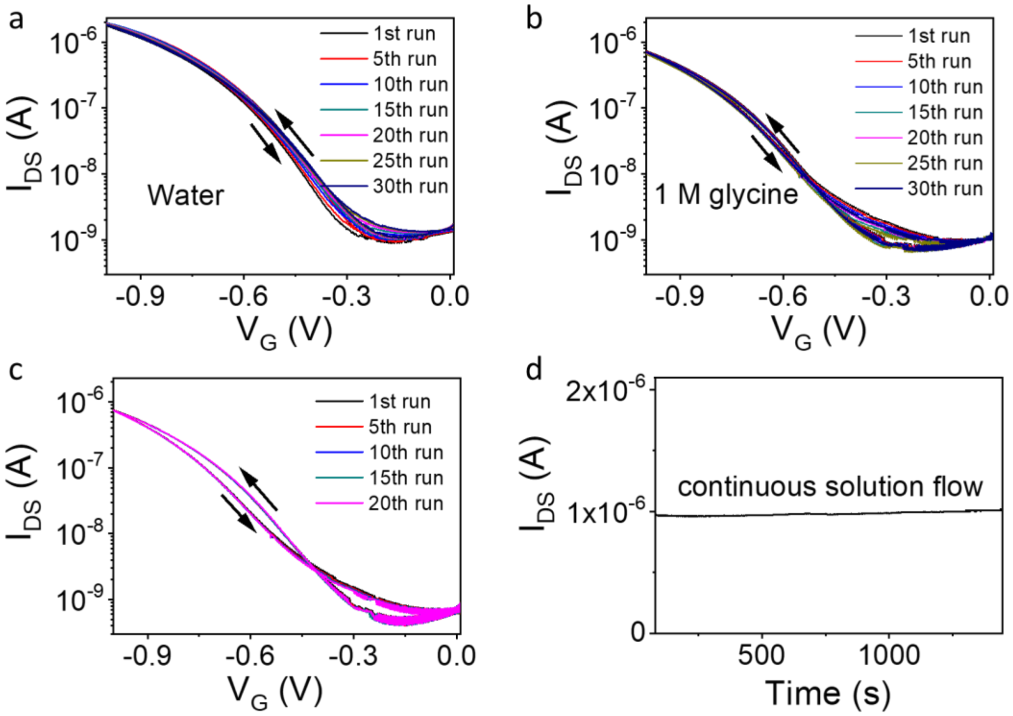

Figure S1. Transfer curves of the EGOFET device characterized with (a) water (30 runs, total time = 2310 s) and (b) 1 M glycine (30 runs, total time = 2310 s) at a flow rate of 100 μL/min in the microfluidic channel. (c) Transfer curves of 1 M glycine after the device with glycine solution inside kept overnight (12 h). (d) I_{DS} (after 80 s stabilization) recorded during the continuous stress measurement with 1 M glycine at a flow rate of 100 μL/min in the microfluidic channel over 1300 s at a fixed V_G of -0.8 V and V_{DS} of -0.7 V.

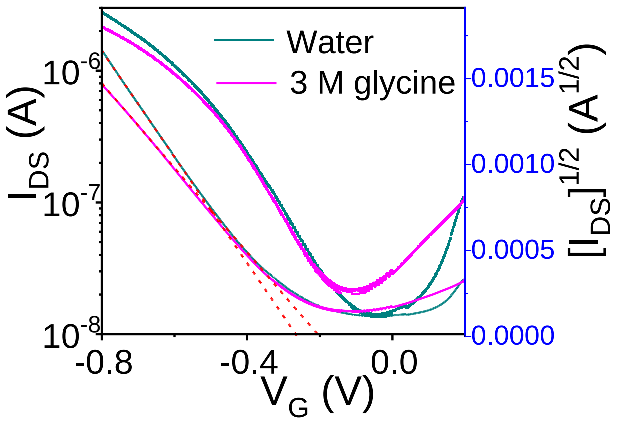

Figure S2. Corresponding transfer characteristics of the devices of water and 3 M glycine for Figure 1b. The red dashed lines show the linear fitting of $(I_{DS})^{0.5}$ vs V_G.

Section S4. Real-time crystallization study from evaporative droplets

Section S4.1. Electrical characterization of the EGOFET

A gold wire with a diameter of 0.5 mm was used as top gate, and was treated with 1H,1H,2H,2H-perfluorodecanethiol (PFDT) to achieve higher hydrophobicity (**Figure S3**) in order to inhibit crystallization at the gate[3]. This was confirmed by eyes looking at the crystallization without Faraday cage, **Figure S4 and movie S1**: the crystals are appearing on the channel region.

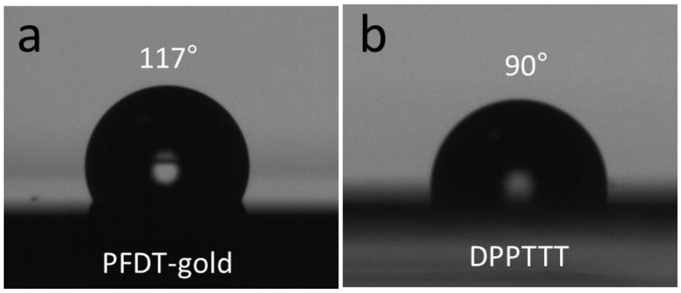

Figure S3: Contact angle measurements of 1 M glycine on (a) PFDT treated gold and (b) DPPTTT substrates.

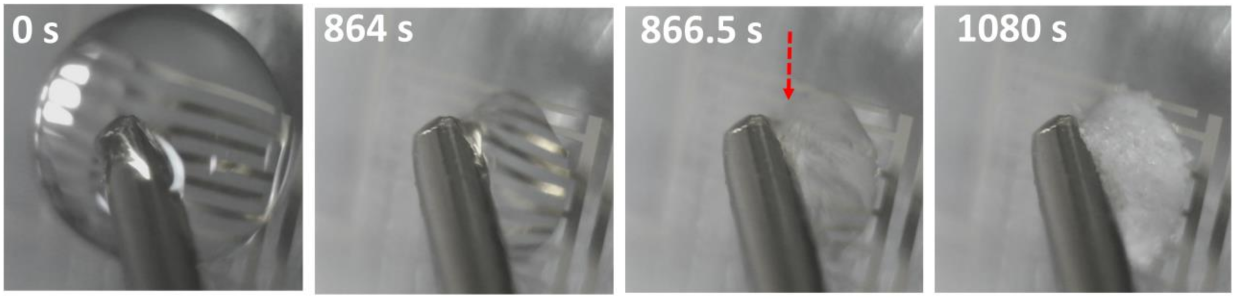

Figure S4. Frames extracted from movie S1 showing the changes in the droplet caused by solvent evaporation and crystallization. Crystals covers the whole surface at ~866.5 s, starting from the contact region with the DPPTTT as indicated by the red dashed arrow.

The transfer and output curves obtained using water and a bare gold wire are shown in **Figure S5 a and 5b**, respectively. The transfer and output curves obtained using water and the PFDT functionalized gold wire are shown in **Figure S5c and 5d**, respectively. The transfer and output curves obtained using 1 M glycine solution and a bare gold wire are shown in **Figure S5e and 5f**, respectively. The transfer and output curves obtained using 1 M glycine solution and the PFDT functionalized gold wire are shown in **Figure S5g and h**, respectively. The use of the PFDT functionalization does not affect the on/off ratio (~100), but one can see that there is a positive shift in V_{Th} and decrease of I_{DS} when the functionalized gold wire is used, as compared to the bare gold (**Figure S5a and 5c**), which can be explained by the potentiometric and capacitive effects by the gate functionalization with PFDT[3]. One can also note that the transfer curve for 1 M glycine solution (**Figure S5e and 5g**) shows small hysteresis for both the bare and PFDT functionalized gold wire gated devices. Overall, the output curves show typical field-effect behavior with no signs of contact resistance.

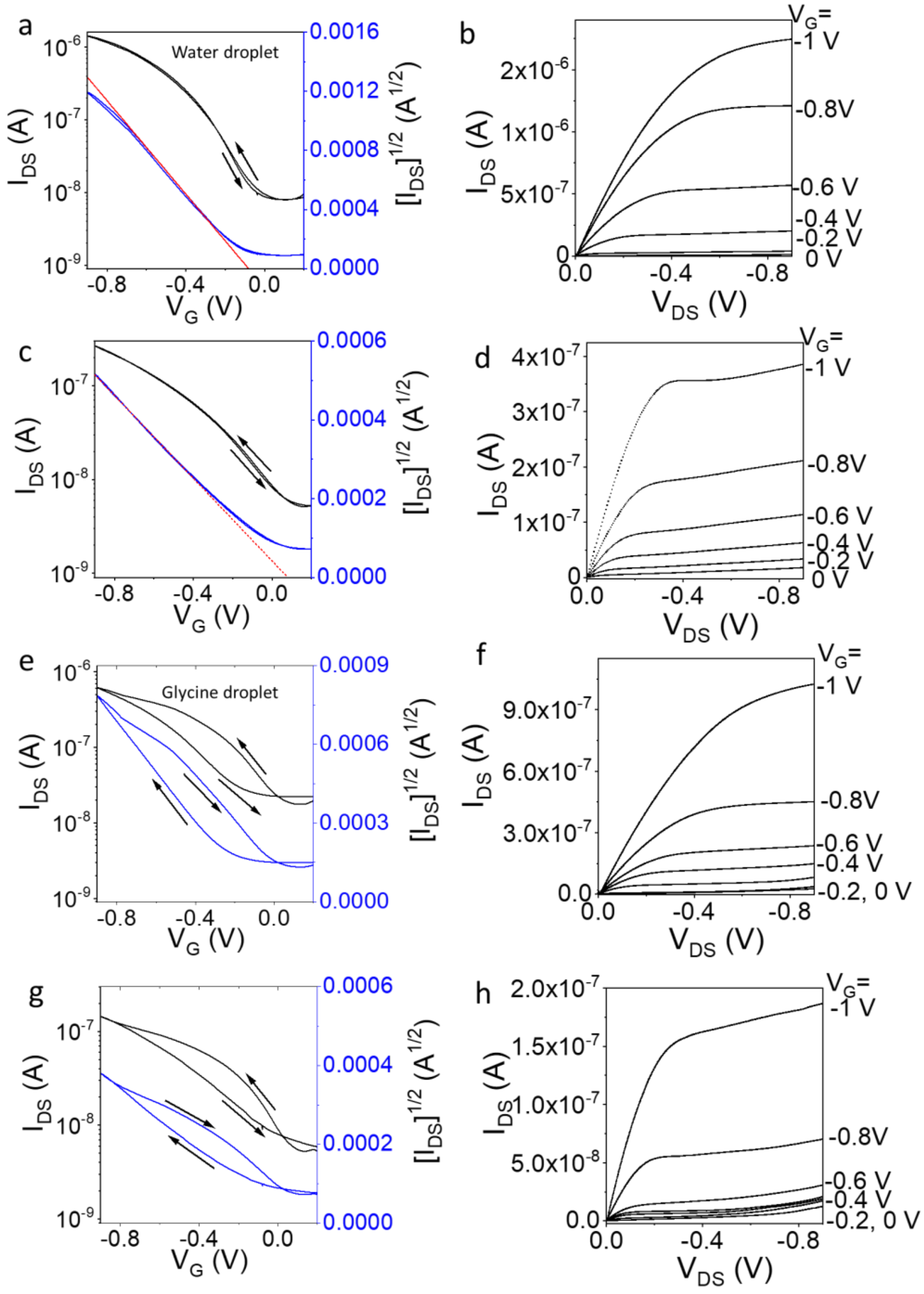

Figure S5. Transfer and output curves using a water droplet using bare gold wire (a, b) and PFDT functionalized gold wire (c, d) as gate electrode. Transfer and output curves using 1 M glycine droplet using bare gold wire (e, f) and PFDT functionalized gold wire (g, h) as gate electrode.

Section S4.2. Calibration measurements

The measurements were performed by drop casting a droplet of solution containing glycine at different concentrations (up to 3M) over the active channel under ambient conditions and at a constant gate voltage (V_G) of -0.8 V and drain-source voltage (V_{DS}) of -0.7 V. A Faraday cage was used to minimize the noise. Representative transfer curves obtained using water and glycine solution are shown in **Figure S6**. At the very beginning, the I_{DS} changes as a result of the formation of EDLs and then stabilize. The average current and its standard deviation, used as error, is taken in the range of 70-90 s for each measurement as in this time the device is shown to be at the equilibrium and crystallization is far from happening.

ΔI is defined as the difference in current at a certain glycine concentration (C_{gly}) with respect to the current measured for water (I_{H2O}). The C_{gly} is approximate to the concentration in electrical double layer when considering that concentration from 0.5 mM to 3 M were explored[4, 5]. **Figure 2b** shows the plot of ($-\Delta I/I_{H2O}$) *vs* C_{gly} in the semi-logarithmic format. An approximate linear trend is seen. The linear fit of the data (red dash line in **Figure 2b**) leads to the following calibration:

$$\frac{-\Delta I}{I_{\mathrm{H2O}}} = 0.0299 + 0.077 \log C_{\mathrm{gly}} \tag{3}$$

with a residual fitting coefficient of 99%.

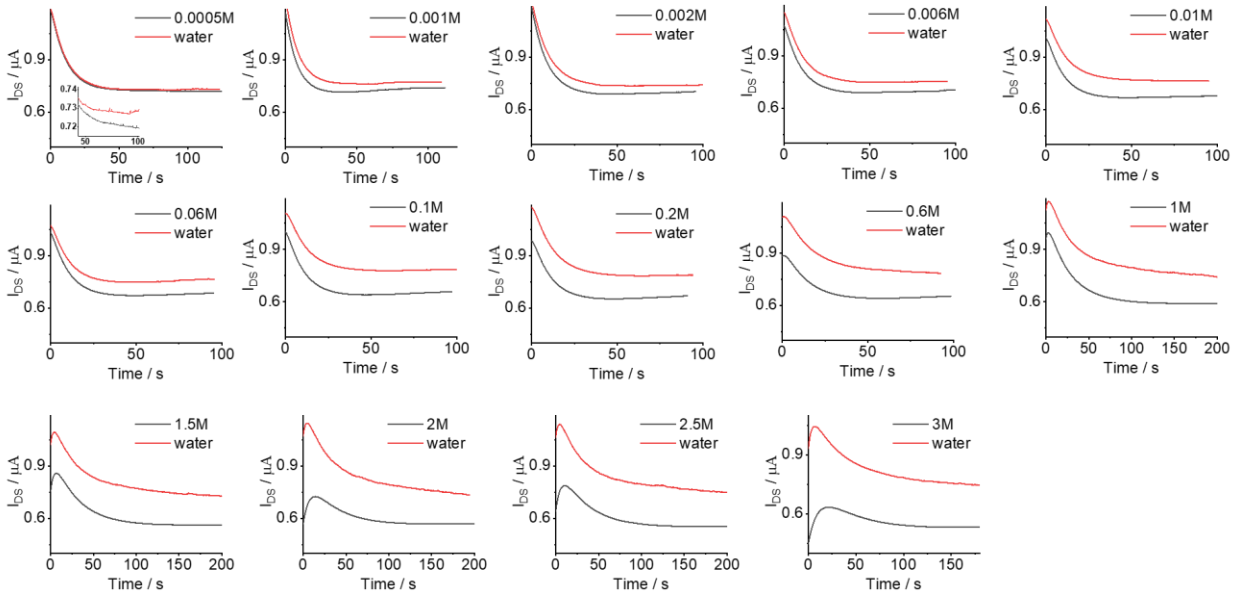

Figure S6. The I_{DS} curves over time measured by drop casting water and glycine solutions at different concentrations (from 0.0005 M to 3 M).

Section S4.3. Real-time measurements

All the experiments included in the main text are performed with a Faraday cage. However, we have also performed some measurements without the cage as this allows to visualize the crystals formation with a camera, by determining the induction time, in addition to the EGOFET recording. The lack of the Faraday cage results in much higher noise level, hence quantitative measurements cannot be performed.

The electrical measurement and the optical image were recorded at the same time once the gold wire was inserted into the droplet (time = 0 s). A V_G of -0.8 V and V_{DS} of -0.7 V were applied. First, a decrease in volume of the droplet due to evaporation was observed, as well as a decrease in the area of the contact with the gold wire (**Figure S4**). The crystals were optically visible at the contact region of droplet with the semiconducting layer after 864 s, and fully covered the surface in just in 1.5 s. The induction corresponds to a sharp increase in the I_{DS} (**Figure S7**), in accordance to previous results obtained with a microelectrode array[6]. Note that this peak in I_{DS} is not observed when water is used as electrolyte and it is seen only when a glycine solution is used as electrolyte in all devices tested, **Figure S8**. The exact shape of the I_{DS} peak may be different in each device, but the sharp increase in current at the induction time is seen in all devices.

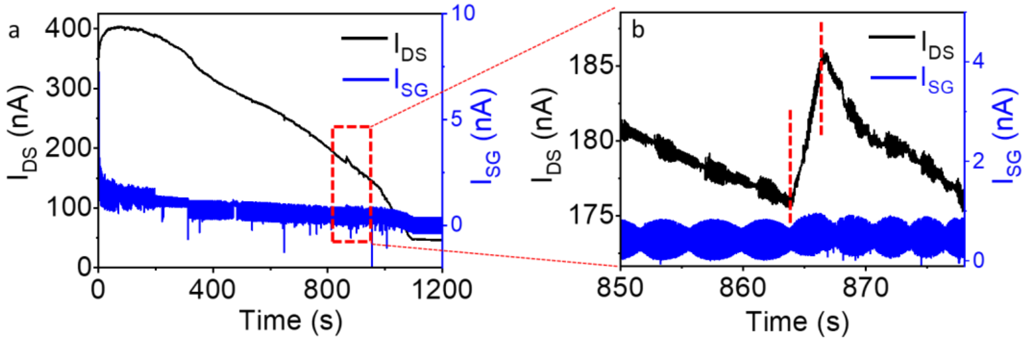

Figure S7. (a) I_{DS} and I_{SG} at fixed V_G (-0.8 V) and V_{DS} (-0.7 V) over the time; (b) enlarged view showing the details of the highlighted red square in (A). Measurements were done without the Faraday cage.

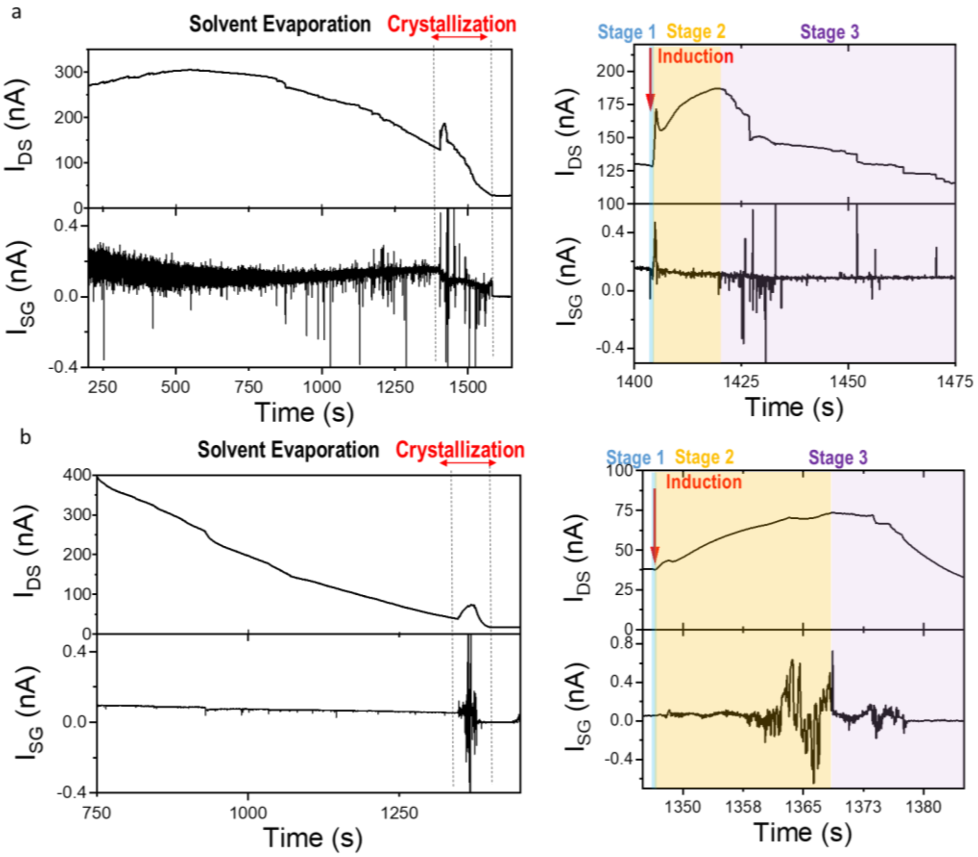

Figure S8. Recorded I_{DS} and I_{SG} over time after drop casting water or 1 M glycine solution at a fixed V_G (-0.8 V) and V_{DS} (-0.7 V) using: (a) device 2 and (b) device 3. Measurements done with the Faraday cage. The right panels are enlarged view related to the changes associated to crystallization. The numbers 1, 2, and 3 remark the different crystallization stages (see main text).

Section S4.4. Stability of the EGOFET device over the crystallization process

To further verify the stability of the EGOFET device and exclude any effect from crystallization on the semiconducting layer, two experiments were carried out.

The first experiment was performed by measuring the conductivity of the semiconducting film by applying a bias voltage of 0.7 V between the source and drain contacts before adding a 3 M glycine droplet (before crystallization) and after washing out the crystals obtained from the crystallization (after crystallization). In between, the droplet was left to evaporate, leading to crystallization until no obvious color change can be observed. As shown in **Figure S9**, the current measured after crystallization recovers to the original value before crystallization. A slow increase of current was observed as a result of the stress measurement, which is commonly seen in organic semiconductors, while the trend is similar for the current curve before and after crystallization.

The second experiment was carried out by measuring the I_{DS} of the EGOFET device by placing water droplet before and after adding a 3 M glycine droplet and leave it to crystallize. As shown in **Figure S10**, the I_{DS} current recovers to the same value after water droplet was used again after removing the glycine crystals.

Therefore, based on the above experiments, it is clear that the EGOFET device is stable over the crystallization process, and there is no effect associated to the presence of the crystals on the semiconducting film.

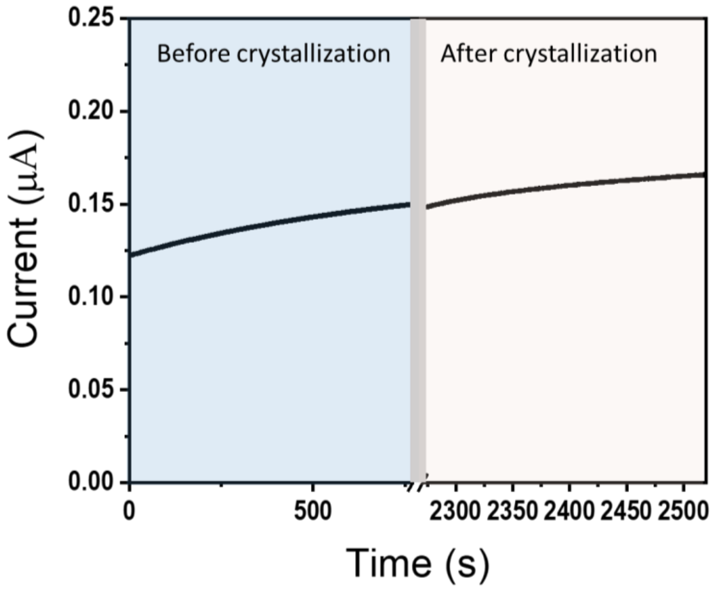

Figure S9. The current curves over time measured at the voltage of 0.7 V before the drop casting of 3 M glycine solution and after washing out the crystals obtained from the crystallization.

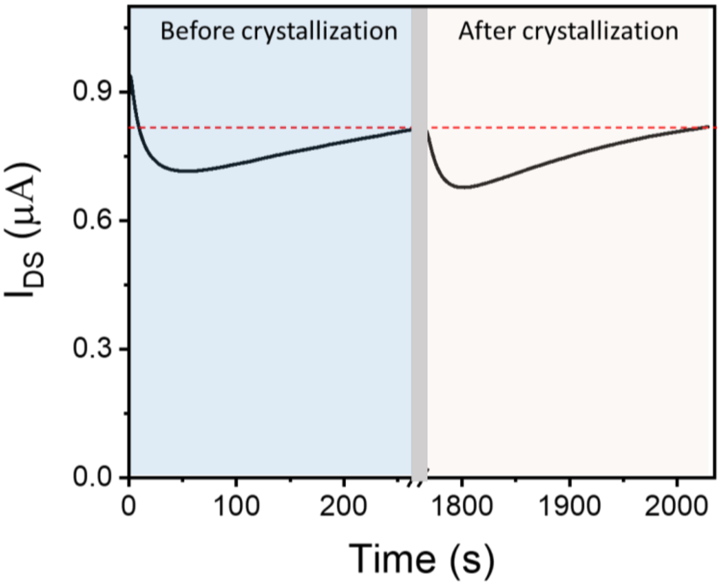

Figure S10. The I_{DS} curves measured by putting water droplet under a constant V_G of -0.8 V and V_{DS} of -0.7 V over time. The grey parts represent the periods that 3 M glycine solution were drop casted and crystallization happen afterward.

Section S5. Calculations of the droplet crystallization dynamics

Section S5.1. Critical concentration and supersaturation ratio

Under the classical nucleation theory, the concentration of molecules must be high enough to reach supersaturation to enable nucleation and crystal growth. In our previous work[6], the critical concentration at which supersaturation is reached was determined from the time at which fluctuations in the recorded current were first observed by using an interdigitated electrode array.

In this study, due to the EGOFET sensitivity, this time can be evaluated more precisely and it can be assigned to the beginning of stage 1 (see main text). The critical concentration of glycine (C_{gly*}) can be calculated as:

$$C_{gly*} = \frac{V_{gly}}{V_{gly} - V_{evap}} C_0 \tag{4}$$

where C_0, V_{gly}, V_{evap} are the initial concentration of glycine, the droplet volume, and the evaporated volume, respectively. As the glycine droplet volume is the same as that of the water droplet (V_{H2O}) in our range of glycine concentrations, the above equation can be re-written as:

$$C_{gly*} = \frac{V_{H2O}}{V_{H2O} - V_{evap}} C_0 = \frac{\sum_0^{t_{H2O}} v_{H2O-i} \times t_i}{\sum_0^{t_w} v_{H2O-i} \times t_i - \sum_0^{t_{nucl}} v_{gly-i} \times t_i} C_0 \approx \frac{t_{H2O}}{t_{H2O} - t_{nucl}} C_0 \tag{5}$$

where t_i is the time calculated from the droplet drop casting, v_{H2O-i} and v_{gly-i} are the evaporation rate at t_i of water droplet and glycine droplet, respectively. t_{H2O} and t_{nucl} are the total evaporation time for water droplet and the time at which stage 1 starts in the case of the glycine droplet. By using t_{H2O} = 1821 s and t_{nucl} accurately determined by the EGOFET measurement, C_{gly*} can be easily derived. From this, one can then calculate the supersaturation ratio (S), as $S = C_{gly*}/C_{sat}$. The results obtained using 3 different devices and the average values are summarized in **Table S1**.

Table S1. Summary of the nucleation time (t_{nucl}), the corresponding critical concentration (C_{gly*}) and supersaturation ratio (S) obtained by drop casting 1 M glycine droplets onto 3 different EGOFETs (Figure 4). S was calculated using C_{sat} of 3.33 M[6].

C_0 / M	t_{nucl} / s	C_{gly*} / M	S
**1 (device 1)**	1432.6	4.7	1.4
**1 (device 2)**	1403.5	4.4	1.3
**1 (device 3)**	1346.2	3.8	1.1
**1 (average)**	1394 ± 44	4.3 ± 0.4	1.3 ± 0.2

Section S5.2. Molecule concentrations and transfer rates

The amount of glycine molecules (N_i) in the EDL at a given time of t_i can be calculated as:

$$N_{\mathrm{i}} = C_{\mathrm{i}}V_{\mathrm{i}} \quad (6)$$

where C_i, V_i are the concentration of glycine molecules and volume of the EDL at t_i, respectively. Assuming the volume of EDL does not change significantly in Stage 1 and Stage 2 of crystallization (see main text), then the following eqs are valid:

$$\frac{\Delta N_j}{N_0} = \frac{N_{\mathrm{i}}-N_0}{N_0} = \frac{C_{\mathrm{i}}V_{\mathrm{i}}-C_0V_0}{C_0V_0} \approx \frac{C_{\mathrm{i}}-C_0}{C_0} = \frac{\Delta C}{C_0} \quad (7)$$

Where ΔC is the relative concentration change in each stage and C_0 is the concentration at the beginning of each stage. Therefore, the transportation rate of glycine molecules in each stage, R_i, can be calculated as the ratio between the total change of the glycine number divided by the time associated to each stage:

$$R_{\mathrm{i}} = \frac{\Delta N_{\mathrm{i}}}{\Delta t} \propto \frac{\Delta \mathrm{C}}{\Delta t} \quad (8)$$

The change in concentration is found by measuring the changes in current and using eq. 3.

To give an example, in the case of device 1 (**Figure 4a**), we have determined $\mathrm{C}_{\mathrm{gly}*}$ to be 4.7 M. This is the concentration of glycine molecules at the beginning of stage 1 (i.e. at 1432.6 s for device 1, main text). The change in current is then correlated to the change in concentration (eq. 7), hence the concentration at the end of stage 1 (C_1) can be calculated.

Eq. 3 can simplified for C_{gly} > 1000 mM to:

$$-\frac{I-I_{\mathrm{H2O}}}{I_{\mathrm{H2O}}} = 0.203 + 3.52 \times 10^{-5} \times C_{\mathrm{gly}} \quad (9)$$

So a change of 1% in current is equivalent to a change of ~28.4% in glycine concentration (in M). In the case of device 1, the change in current over stage 1 is: (96.2-97.2)/97.2 = -1.0% and the

initial concentration C_{gly*} is 4.7 M, so the concentration at the end of stage 1, C_1, is ~6.0 M. As stage 1 takes 300 ms, R_1 is (6.0-4.7) M/0.3 s = 4.3 M/s. Table S2 reports all values used.

By using the same approach, we can also get the concentration at the end of stage 2 (C_2). In this case, the current change is (141.3-96.2)/96.2 = 46.9%, which is larger than the change observed for glycine concentrations in the range 0.5 mM - 3 M. Using eq. 3, we obtain $C_2 = 5x10^{-6}$ M, which is well below the minimum concentration that we can detect with the EGOFET (**Figure 2b**). Assuming that the concentration of molecules at the end of Stage 2 is so small to be approximated to zero, compared to the initial concentration, then a change in concentration of (0-6.0) M = -6.0 M is measured over Stage 2, which gives R_2 = -6.0 M/14.9 s= -0.4 M/s. The above calculations are also applied to the other two devices. All values are summarized in **Table S2**.

Note that the water evaporation rate is calculated to be 2 μL/1821 s (S4.1), and the glycine concentration changes due to evaporation from stage 1 to 2 for device 1 (**Figure 4a**) can be calculated to be 0.016% and 0.8% respectively, which is negligible when compared to the concentration change (28.4% and 100% for stage 1 and 2 as shown in **Table S3**) due to crystallization. Thus, in all the calculations for the concentrations and transfer rates, the concentration changes ascribed to water evaporation are ignored.

Table S2. A summary of the characteristic times, I_{DS}, relative current change (-ΔI), and related molecules concentration (C_i) at the beginning and end of Stages 1 and 2 (extracted from Figure 3 and Figure S8).

	Time / s	I_{DS} / nA	-ΔI / nA	Δt / s	C_i / M
**Device 1**					
Beginning Stage 1	1432.6	97.2			4.7
End stage 1/Beginning Stage 2	1432.9	96.2	1.0	0.3	6.0
End Stage 2	1447.8	141.3	-45.1	14.9	<<0.0005
**Device 2**					
	Time / s	I_{DS} / nA	-ΔI / nA	Δt / s	C_i / M
Beginning Stage 1	1403.5	129.0			4.4
End Stage 1/ Beginning Stage 2	1404.1	128.2	0.8	0.6	5.2
End Stage 2	1420.0	187.1	-58.8	15.9	<<0.0005
**Device 3**					
	Time / s	I_{DS} / nA	-ΔI / nA	Δt / s	C_i / M
Beginning Stage 1	1346.20	38.0			3.8
End Stage 1/ Beginning Stage 2	1346.50	37.6	0.4	0.3	5.0
End Stage 2	1368.50	73.8	-36.2	22.0	<<0.0005

Table S3. Quantitative analysis of the crystallization dynamics. Table reporting the relative I_{DS} change ($-\Delta I/I_0$), the relative concentration change ($\Delta C/C_0$) from the beginning to the end of each stage and the corresponding molecular transport rate (R) calculated for each stage, as extracted from Figure 3 and Figure S8. Measurements have been performed on 3 different devices.

		**Device 1**	**Device 2**	**Device 3**	**Average**
**Stage 1**	$-\Delta I/I_0$ (%)	1.0	0.6	1.1	0.9 ± 0.3
	$\Delta C/C_0$ (%)	28.4	17.0	31.2	25.5 ± 7.5
	R_1 (M s^{-1})	4.3	1.3	4.0	3.2 ± 1.7
**Stage 2**	$-\Delta I/I_0$ (%)	-46.9	-45.9	-96.3	-63.0 ± 28.8
	$\Delta C/C_0$ (%)	-100	-100	-100	-100
	R_2 (M s^{-1})	-0.4	-0.3	-0.2	-0.3 ± 0.1

Section S6. Movie S1

Movie taken with a camera mounted on the optical microscope showing the real time monitoring of glycine crystallization from solution.

References

1. Gundlach, D. J.; Royer, J. E.; Park, S. K.; Subramanian, S.; Jurchescu, O. D.; Hamadani, B. H.; Moad, A. J.; Kline, R. J.; Teague, L. C.; Kirillov, O.; Richter, C. A.; Kushmerick, J. G.; Richter, L. J.; Parkin, S. R.; Jackson, T. N.; Anthony, J. E., Contact-induced crystallinity for high-performance soluble acene-based transistors and circuits. *Nat. Mater.* **2008,** *7* (3), 216-221.

2. Kim, S. H.; Hong, K.; Xie, W.; Lee, K. H.; Zhang, S.; Lodge, T. P.; Frisbie, C. D., Electrolyte-gated transistors for organic and printed electronics. *Adv. Mater.* **2013,** *25* (13), 1822-46.

3. Leonardi, F.; Tamayo, A.; Casalini, S.; Mas-Torrent, M., Modification of the gate electrode by self-assembled monolayers in flexible electrolyte-gated organic field effect transistors: work function vs. capacitance effects. *RSC Adv.* **2018**, *8* (48), 27509-27515.

4. Miyashita, S.; Komatsu, H.; Suzuki, Y.; Nakada, T., Observation of the concentration distribution around a growing lysozyme crystal. *J. Cryst. Growth* **1994**, *141* (3-4), 419-424.

5. Finney, A. R.; McPherson, I. J.; Unwin, P. R.; Salvalaglio, M., (2021). Electrochemistry, Ion Adsorption and Dynamics in the Double Layer: A Study of NaCl (aq) on Graphite. *Chem. Sci.* **2021**, *12* (33), 11166.

6. Tong, J.; Doumbia, A.; Turner, M. L.; Casiraghi, C., Real-time monitoring of crystallization from solution by using an interdigitated array electrode sensor. *Nanoscale Horiz.* **2021**, *6* (6), 468-473.
